# Primary stability of nailing versus low-profile dual plating of mid-clavicular fractures– a biomechanical cadaveric study

**DOI:** 10.1007/s00068-025-02854-2

**Published:** 2025-04-29

**Authors:** Fabian Pretz, Ivan Zderic, Frank J. P. Beeres, Björn-Christian Link, Reto Babst, Yannic Lecoultre, Boyko Gueorguiev, Peter Varga, Torsten Pastor, Bryan J. M. van de Wall

**Affiliations:** 1 AO Research Institute Davos, Davos, Switzerland; 2 Department of Orthopaedic and Trauma Surgery, Lucerne Cantonal Hospital, Lucerne, Switzerland; 3 Department of Health Science and Medicine, University of Lucerne, Lucerne, Switzerland; 4 Medical Faculty, University of Zurich, Zurich, Switzerland

**Keywords:** Midshaft clavicle fractures, Dual plating, Intramedullary fixation, TEN, Biomechanics, Motion tracking

## Abstract

**Introduction:**

Low-profile dual plating techniques have gained popularity for diaphyseal clavicula fractures due to their potential to reduce soft tissue irritation. Intramedullary nailing is also an established surgical option for treatment of diaphyseal clavicle fractures. The present study therefore aimed to evaluate whether a 2 × 2.0 mm dual plating system can achieve biomechanical performance comparable to intramedullary nailing while reducing implant-related complications.

**Methodology:**

Twelve paired human cadaveric clavicles with simulated unstable diaphyseal shaft fractures AO/OTA 15.2 C were stabilized via elastic nailing (Group 1) or dual plating using a superior and an anterior 2.0 mm plate (Group 2). Specimens underwent biomechanical testing with initial quasistatic superior-inferior and anterior-posterior bending, followed by cyclic superior-inferior loading to failure. Interfragmentary movements were monitored by optical motion tracking.

**Results:**

Dual plating demonstrated significantly higher initial construct stiffness in all bending directions and a reduced neutral zone compared to intramedullary nailing (*p* ≤ 0.016). In addition, fracture displacement amplitudes over all cycles were significantly higher in Group 1 versus Group 2 (*p* = 0.002). The number of cycles required to reach the test endpoint at a 45 mm displacement did not differ significantly between the groups (*p* = 0.160), with Group 1 averaging 24,420 cycles (SD ± 3,615) and Group 2 averaging 28,232 cycles (SD ± 5,417).

**Conclusion:**

Low-profile dual plating may offer improved initial stability of midshaft clavicle fractures without compromising their long-term performance, making it a promising alternative to elastic nailing. In selected patients with simpler unstable midshaft clavicle fractures, 2 × 2.0 mm dual plating may offer effective biomechanical stability.

## Introduction

In recent decades, the incidence of operative interventions for clavicle fractures has significantly increased [1]. This increase can be attributed, among other factors, to the often unsatisfactory outcomes and up to 32% non-union rates of conservatively treated displaced fractures [1–5]. The majority of clavicle fractures affect the diaphyseal region (69–82%), while the medial and lateral ends gain additional stability from strong ligamentous and muscular structures (6). Plate osteosynthesis ensures stable fixation and high rotational stability but is often associated with complications such as soft tissue irritation and hypertrophic scarring. Furthermore, high implant removal rates of up to 64% have been reported with traditional 3.5 mm superiorly positioned plates [7]. Alternatively, intramedullary stabilization by means of a non-interlocked nail offers a less invasive method that spares soft tissue, although it does not ensure rotational stability and can also lead to complications such as implant migration [4, 7, 8]. Clinical studies have shown that intramedullary fixation frequently yields good results with low non-union rates, although in 50% of cases, an open reduction is necessary [9]. However, there is an elevated risk of refracture with plate osteosynthesis compared to intramedullary fixation [10].

To minimize complications and reduce the need for implant removal, osteosynthesis is increasingly being optimized with low-profile dual plating, a trend supported by biomechanical studies demonstrating enhanced stability compared to single plates [4, 11, 12]. Additionally, Titanium Elastic Nail (TEN) often necessitates fracture site exposure for reduction and can result in nail migration, frequently requiring implant removal [4, 7, 8]. Hence, there is a demand for an low profile implant that provides at least the same stability as TEN, does not migrate, and causes minimal soft tissue irritation. In a biomechanical study, dual plating using low-profile 2 × 2.0 mm matrix mandible plates or a combination of a 2.5 mm anterior and a 2.0 mm superior plate, was compared to a superior 3.5 mm clavicular locking compression plate (LCP) [13]. The results showed a significant disadvantage for the 3.5 mm clavicle plate regarding displacement under compressive load compared to both dual plating configurations. However, the number of cycles to failure was significantly higher for the 3.5 mm plate compared to the 2 × 2.0 mm dual plating.

Another biomechanical study evaluated the same 2.5/2.0 mm low-profile dual plating configuration in comparison to a 2.7 mm superior variable angle (VA) LCP clavicle plate using human clavicles [12]. The dual plating configuration demonstrated significant advantages for initial construct stiffness, achieved smaller fracture gap displacement and higher number of cycles to failure. The authors of these studies concluded that dual plating with 2.5/2.0 mm plates represented a biomechanically safe alternative to conventional plate osteosynthesis for diaphyseal midshaft clavicle fractures. However, the 2.5 mm anterior plate may still cause soft tissue irritation due to its thickness, prompting the question of whether reducing plate thickness could offer a viable alternative to TEN [9, 11, 12, 14]. While intramedullary nailing may reduce soft tissue irritation, its biomechanical stability compared to low-profile dual plating remains uncertain, supporting the rationale for investigating 2 × 2.0 mm plates as a potential alternative for treatment of simple midshaft clavicle fractures.

Therefore, the aim of the current study was to compare the biomechanical competence of 2 × 2.0 mm low-profile dual plating versus 2.5 mm TEN fixation of simple midshaft clavicle fractures in a human cadaveric model.

## Materials and methods

### Specimens & study groups

Twelve paired, fresh-frozen (-20 °C) human cadaveric clavicles from two male and four female donors, with a mean age of 82.2 years (standard deviation (SD): ± 7.86 years) without injuries or prior surgeries to the clavicle were used. All donors gave their informed consent inherent within the donation of the anatomical gift statement during their lifetime. The sample size was calculated based on a priori power analysis, assuming that after biomechanical testing 30% mean difference in fracture displacement between the groups would represent a clinically meaningful difference, and that a standard deviation of 66% in each group (effect size: 0.3; SD: 0.2) could be expected. Based on these assumptions, a sample size of 6 specimens would be necessary to reach significant differences between the two groups. Specimens were thawed at room temperature and subsequently scanned via quantitative computed tomography at a slice thickness of 0.63 mm (Revolution EVO, GE Medical Systems AG, Switzerland) to determine trabecular bone density of each clavicle in the mid-diaphyseal region. A phantom calibration (European Forearm Phantom QRM-BDC/6, QRM GmbH, Möhrendorf, Germany) was employed for bone mineral density (BMD) calculation. Clavicles were entirely stripped of soft tissues and assigned pairwise to two equally sized groups (*n* = 6) for instrumentation via intramedullary nailing or dual plate osteosynthesis, with each group having evenly distributed right and left clavicles.

## Surgical techniques

**Fig. 1:**
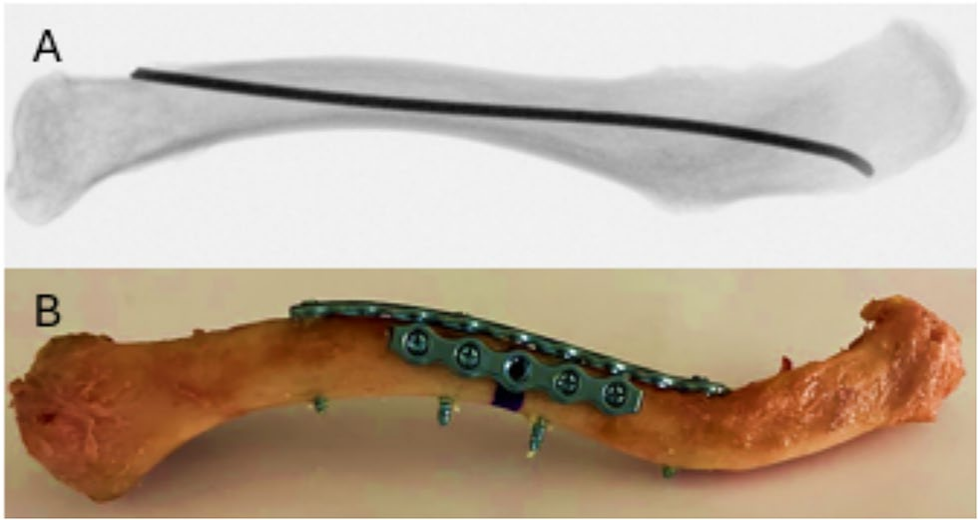
X-ray of a left clavicle instrumented with TEN **A**) and photograph of a left clavicle instrumented via 2 × 2.0 mm dual plating with the planned osteotomy marked with black ink (**B**)

In Group 1, an intramedullary nail (TEN, 2.5 mm diameter; length 440 mm; Johnson & Johnson MedTech, Zuchwil, Switzerland) made of titanium alloy was inserted via a medial entry point 15 mm lateral to the medial clavicular end. The anterior cortex was opened using an awl, and the nail was introduced laterally through the intramedullary canal using a T-handle. The curved tip of the nail facilitated navigation, and no additional bending was required since the nail conformed to the intramedullary canal. The nail was advanced laterally to the tuberculum conoideum of the clavicle without penetrating the cortex (Fig. 1A), and its positioning was confirmed with C-arm imaging (Ziehm Vision RFD, Nürnberg, Germany). The medial end of the nail was trimmed to leave a 5 mm projection.

In Group 2, the specimens were instrumented with a superiorly placed 5-hole 2.0 mm, and an anteriorly placed 9-hole 2.0 mm matrix mandible plate, both cut from a 20-hole titanium alloy plate (Johnson & Johnson, Zuchwil, Switzerland) due to the unavailability of pre-cut lengths. Only the anterior plates required precontouring to fit to the clavicles’ anatomy. Each plate was positioned with the middle hole over the planned osteotomy gap– the third hole of the 5-hole plate and fifth hole of the 9-hole plate– and temporarily fixed to the specimen using clamps. Pilot holes were drilled bicortically using a 1.8 mm drill bit. In the 5-hole superior plate, screw hole positions 1, 2, 4, and 5, counted from medial, were used for fixation, whereas in the anterior plate, positions 1, 4, 6, and 9 were considered. Plates were secured with 2.4 mm bicortical locking screws (Fig. 1B). All screws were tightened based on the operator´s tactile feedback.

The complete instrumentation procedure was performed by an experienced surgeon following the surgical guides of the used implants.

Following the instrumentation in each group, a 5 mm-wide osteotomy was set at the midshaft of each clavicle using an oscillating saw, simulating an unstable AO/OTA 15.2 C diaphyseal fracture. This fracture model was adopted from previous biomechanical studies [12, 13]. Only the cortex was cut in Group 1, with excess bone material removed using a Luer to protect the osteosynthesis material and in both groups special care was taken to avoid damage of the implants. The medial and lateral ends of the clavicles were embedded in collinear polymethyl methacrylate (PMMA; SCS-Beracryl D28; Suter Kunststoffe AG, Fraubrunnen, Switzerland) cylinders, with a consistent distance of 100 mm between medial and lateral PMMA end for uniform conditions in the subsequent biomechanical testing [12]. The medial end of the nail was covered with modeling clay during the embedding process to ensure that the TEN was not embedded in PMMA. Optical markers were attached to each clavicular fragment for motion tracking.

## Biomechanical testing

Each specimen underwent four different tests. Initially, a non-destructive quasistatic bending tests in anterior-posterior (Test 1) and superior-inferior (Test 2) directions were conducted. Subsequently, a cyclic test in superior-inferior bending direction was performed (Test 3). Finally, a cyclic test in superior-inferior bending direction with progressively increasing loading to catastrophic failure of the osteosynthesized clavicle was conducted (Test 4). The biomechanical testing protocol followed established methodologies, ensuring comparability with previous studies [12, 13].

**Fig. 2:**
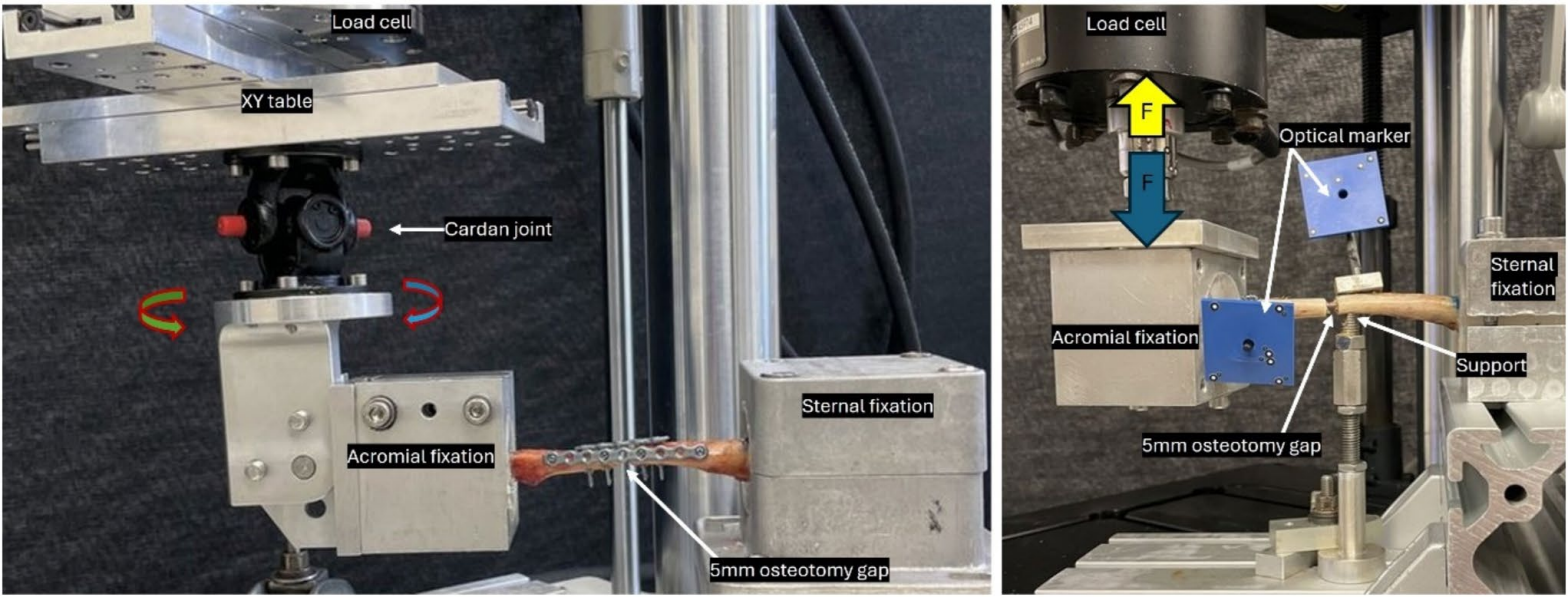
Setup with a left specimen for quasistatic bending test (left) and cyclic loading test (right)

Test 1 was performed on a servo-hydraulic test machine (Mini Bionix II 858, MTS Systems Corp., Eden Prairie, MN, USA), equipped with a 4 kN load cell. The clavicle was fixed at its sternal end to the machine base (Fig. 2left). Fixation was achieved using PMMA to ensure a stable connection between the fixation points without affecting the osseous structures. The acromial end of the clavicle was attached to the machine actuator, which was connected to the load cell via an XY table. This configuration balanced shear and bending forces. Using this setup, anterior-posterior bending tests were performed to simulate the forces acting on the clavicle during shoulder movements. Test 2 employed the same setup, however, the specimen was rotated by 90° to impose superior-inferior loading. The loading protocol for Tests 1 and 2 was defined as a non-destructive quasistatic increase of the compression for up to 30 N at a rate of 5 N/s.

Tests 3 and 4 were cyclic bending tests in the superior-inferior direction, conducted on an electrodynamic test machine (MTS Acumen; MTS Systems Corp., Eden Prairie, MN, USA) equipped with a 3 kN load cell (Fig. 2right). The test setup was based on previous studies [12, 13] and modified to address the susceptibility of clavicle nails to rotational forces, as observed in prior research [15]. To mitigate this effect, rigid fixation was implemented. The sternal end of the clavicle was fixed to the machine base and the acromial embedding was attached to the machine actuator using custom holders. Additionally, a pin was placed under the sternal fragment to provide medial support and enhance the stability of the medial fragment. Test 3 involved a cyclic loading with a constant amplitude between 50 N compression and 20 N tension at 2 Hz for 20,000 cycles [12, 13]. In the final Test 4, the starting peak loads were equivalent to those of Test 3 and were then progressively increased cycle by cycle at 0.05 N/cycle each until the osteosynthesized clavicle failed catastrophically [12, 13]. The test was terminated when an axial displacement of 45 mm was reached, which was defined as failure [12].

## Data acquisition and analysis

Machine data in terms of as axial force and axial displacement were continuously recorded during the test at a rate of 32 Hz. Bending stiffness in anterior-posterior and superior-inferior and neutral zone (NZ) of the various constructs were calculated from the angle-torque curve within the quasistatic range between 1.5 and 2.5 Nm. The neutral zone represents the range of motion where the osteosynthesis construct provides minimal resistance, indicating the initial compliance of the fixation. It serves as a critical parameter for assessing stability, as a smaller neutral zone suggests higher primary stability.

The coordinates of the markers attached to the bone fragments during cyclic testing were continuously captured throughout the cyclic tests at 25 Hz using non-contact full-field stereographic camera-based motion tracking technology (Aramis SRX; GOM Zeiss Metrology GmbH, Braunschweig, Germany), enabling evaluation of fragment movements in all six degrees of freedom. Using motion tracking data, fracture displacement was defined and assessed as the relative movement between the two fragments measured in a superoinferior direction at the most inferior point of the fracture gap.

Fracture displacement amplitude was analyzed as the difference between peak values in compression and tension, at equidistant time intervals corresponding to 2,500, 5,000, 7,500, 10,000, 12,500, 15,000, 17,500, and 20,000 cycles.

Statistical analysis among the parameters of interest was conducted using R software (Version 4.x; R Foundation for Statistical Computing, Vienna, Austria). The Shapiro-Wilk test was conducted to assess and prove normality of data distribution. Significant differences between the groups for fracture displacement measured over the different time intervals was analyzed with General Linear Model (GLM) Repeated Measures (RM) test. Significant differences between the groups with regard to initial stiffness, NZ and cycles to failure were determined with a paired t-test. Level of significance was set at 0.05.

## Results

### Bone mineral density

The analysis of mean BMD revealed a density of 194.87 mgHA/cm³ (SD ± 38.10) in Group 1, and a density of 194.75 mgHA/cm³ (SD ± 44.86) in Group 2, showing no significant difference between the two groups (*p* = 0.991).

## Initial stiffness and neutral zone

The values for initial stiffness are summarized in Table 1 for each group and loading direction separately. Dual plating was associated with significantly higher values versus TEN fixation for each parameter (*p* ≤ 0.005). The neutral zone also exhibited significant differences between the two groups (*p* ≤ 0.016).

**Table 1:** Initial stiffness during anterior, posterior, superior, and inferior bending, and neutral zone in anterior-posterior and superior-inferior directions, shown for the two study groups separately in terms of mean value ± standard deviation, together with the corresponding p-value denoting significant difference between the two groups

Parameter and loading direction	TEN	Dual Plate	*p* value
Stiffness [N/mm]			
Anterior	0.07 ± 0.01	0.61 ± 0.15	<0.001
Posterior	0.08 ± 0.02	0.63 ± 0.17	0.001
Superior	0.07 ± 0.03	0.53 ± 0.21	0.005
Inferior	0.08 ± 0.02	0.53 ± 0.21	0.005
Neutral Zone [deg]			
Anterior-Posterior	4.47 ± 0.69	1.66 ± 0.63	0.001
Superior-Inferior	4.54 ± 0.74	1.75 ± 0.85	0.016

## Fracture displacement

**Fig. 3:**
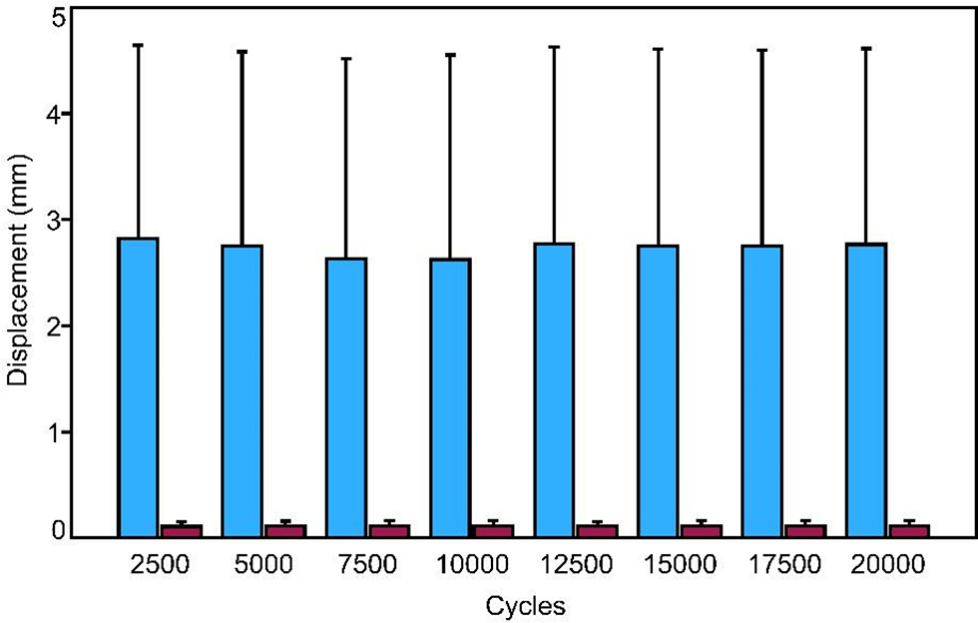
Shear fracture displacement amplitude shown at intermittent time points every 2500 cycles over the first 20,000 cycles in terms of mean value and standard deviation for each group separately

Figure 3 shows the shear fracture displacement at intervals of 2,500 cycles between Group 1 and Group 2. TEN constructs were associated with significantly higher values compared to dual plating (*p* = 0.002).

### Cycles to failure

The number of cycles to failure remained without significant difference between the groups (*p* = 0.16). Group 1 reached an average cycle count of 24,420 (SD ± 3,614.65), while Group 2 exhibited an average cycle count of 28,232 (SD ± 5,417.13).

### Failure modes

**Fig. 4:**
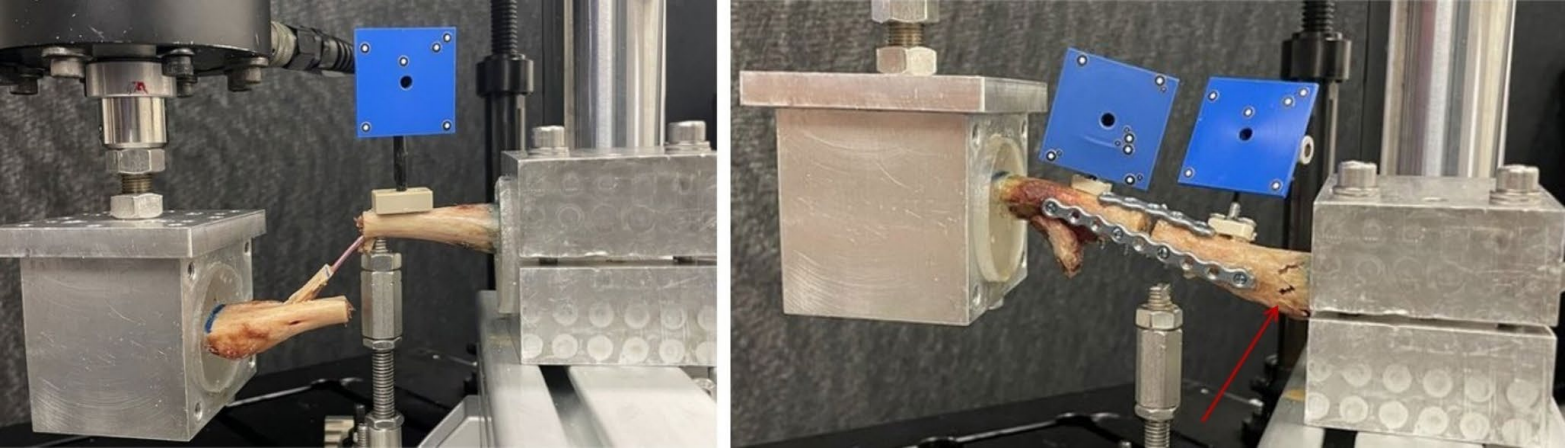
Failure modes observed in Group 1 (left) and Group 2 (right) after cyclic testing

In Group 1, an expansion of the intramedullary canal was observed in each specimen. Additionally, three of the specimens experienced a fracture at the junction between the acromial PMMA and the clavicle. In two cases, a portion of the superior cortex fractured (Fig. 4). No nail breakage was observed.

In Group 2, there was no failure or loosening of the osteosynthesis material. Each specimen experienced a bone fracture at the transition from the PMMA to the lateral clavicle.

## Discussion

This study investigated the biomechanical stability of intramedullary nail fixation (TEN) compared to low-profile dual plating (2 × 2.0 mm) of unstable midshaft clavicle fractures using human cadaveric specimens. Currently, open reduction and internal fixation (ORIF) using single or dual plating or intramedullary nailing are the most common surgical techniques for treating diaphyseal clavicle fractures [4, 9, 14, 16].

The results of this study show significantly higher biomechanical stability of dual plating compared to intramedullary nailing, especially in terms of initial stiffness and shear displacement of the lateral fragment relative to the medial fragment. These stability advantages of dual plating are consistent with the findings by Pastor et al. (2022), who also demonstrated improved initial stiffness in dual-plating procedures for treatment of clavicle fractures. However, the authors compared two dual plating configurations (2.0/2.0 mm and 2.5/2.0 mm) with conventional 3.5 mm LCP superior plating, whereas the present study compared dual plating with TEN fixation, thereby providing a different perspective on biomechanical stability in fixed clavicle fractures [13].

Despite the advantage in terms of lower invasiveness, intramedullary nailing has limitations, particularly due to a lower stability, as underscored by our tests. Previous biomechanical studies have highlighted TEN’s susceptibility to shear movements, which can have adverse effects on unstable fracture patterns [15, 17]. These shear movements can impair interfragmentary callus formation and increase the risk of complications, making TEN more suitable for simpler diaphyseal fractures with bone fragment contact to reduce rotational forces [14, 18]. However, differences in non-union rates between intramedullary nailing and plate osteosynthesis could not be confirmed in a meta-analysis [14]. A meta-analysis of randomized controlled trials demonstrated that intramedullary nailing offers advantages for diaphyseal clavicle fractures, particularly in terms of reduced operative time, faster fracture healing, and a lower infection rate. Due to the TEN design and the lack of anchoring options, it does not provide adequate rotational stability and, therefore, merely serves as a splint for the clavicle without ensuring fixation of the fracture fragments relative to each other [15]. Additionally, an overly large medullary canal combined with an inadequately chosen TEN diameters can lead to instability, increasing the risk of clinical fixation failure. Particularly, an incorrect indication for TEN application in unstable B and C fractures according to the AO/OTA classification, where mutual support of the fragments is lacking, may result in rotation of the clavicle or its fragments [14, 18–20]. This rotational instability is particularly evident during shoulder abduction beyond 80° [21].

By using a minimally invasive technique, TEN offers clear benefits regarding reduced soft tissue irritation and may be suitable for patients who prioritize tissue preservation. However, previous studies have reported high rates of open reduction with TEN, reaching up to 50% of cases [9]. Although open reduction facilitates a less invasive procedure by eliminating the need for extensive preparation and improving instrument access, it partially offsets the advantages of reduced invasiveness.

Another relevant aspect is fixation in younger, athletically active patients, as TEN is often used in this group. However, the lower stiffness of TEN, combined with repeated loads, may lead to early loosening, as suggested by our cyclic tests. The low bone mineral density of the older cadaveric specimens in our study could have influenced the results, and further research is needed to validate the findings for younger populations.

The 2 × 2.0 mm dual low profile plate osteosynthesis demonstrated biomechanical superiority in our investigation and could represent a viable alternative to the conventional single plating [11, 13]. The meta-analysis and systematic review by Rompen et al. (2022) demonstrated a less complications and lower reoperation rates in the dual plating group, with the smallest plate profile utilized in the analyzed dual plating methods being a 2.4/2.0 mm construct. The application of the 2 × 2.0 mm dual plating method represents a departure from conventional approaches. Biomechanical evidence indicates that this construct is significantly less stable compared to standard 3.5 mm plates [13]. Consequently, the selection of appropriate indications for this technique is of paramount importance, particularly given the current lack of clinical data supporting its use and its mechanical inferiority relative to established methods.

Clinical studies have demonstrated that the need for hardware removal in the treatment of clavicle fractures with low-profile dual plating can be reduced due to fewer occurrences of implant irritation [22, 23]. The meta-analysis by You et al. (2021) also demonstrated a 3.9-fold higher removal rate for the single plate fixation method compared to the dual plating method [24]. However, it should be noted that these studies utilized different plate sizes, including 2.4/2.0 mm, 2.4/2.4 mm, and 2.7/2.4 mm, which differ from the constructs investigated in our study. The present findings support the notion that the dual plating technique is a safe and effective alternative. Unlike TEN, the stiff fixation reduces the risk of shear and rotational forces, which may positively impact fracture stability and healing.

This study has several limitations. First, the use of a 2.5 mm TEN without adapting it to the varying diameters of the intramedullary canals in the cadaveric specimens may have biased the results. Previous studies have shown that adapting the nail thickness to the individual intramedullary canal enhances axial stability [17, 21]. Similarly, rotation forces tested in a controlled environment could yield more realistic results, as rotation is an essential load component in vivo, while the medial and lateral ends of the clavicle could gain stability through ligamentous and muscular structures (6). However, this approach was selected to ensure consistency and comparability by using uniform implants. Another limitation of this study is the advanced age and low BMD of the cadaveric specimens used. TEN is typically preferred for younger patients with higher bone strength, which may affect the transferability of the results. Particularly in the failure load test, no material failure of the osteosynthesis was observed; instead, fractures occurred in the bone at the transition zone between PMMA and bone. This prevented a conclusive evaluation of the osteosynthesis material under failure conditions. Further biomechanical analyses using bone specimens with higher BMD values are necessary to realistically simulate the preferred use of TEN in younger patients. While the present findings generally support the use of double plate osteosynthesis for clavicle fractures requiring high biomechanical stability and precise rotational control, they should first be thoroughly analyzed in clinical studies with more stable fracture patterns and carefully selected patient groups (i.e., cooperative non-smokers with good bone quality). Meanwhile, minimally invasive TEN use offers advantages for simple fractures, where tissue preservation and aesthetic considerations are prioritized, but it is unsuitable for complex fractures with a large fracture gap, as observed in the present study [18, 20]. The selection of this worst-case fracture type in the present study was based on applying the maximum possible load to the osteosynthesis material while also allowing for a comparison with previous biomechanical studies [12, 13]. However, the simulated C fracture with this fracture gap is not optimal for a biomechanical comparison of the two fixation methods in this study.

Future biomechanical research should utilize simulated fractures that better align with the indications of the fixation method. Only then should clinical studies involving younger patient populations be conducted to further validate the findings and to investigate factors such as BMD and postoperative loading patterns in greater detail. Both methods have specific advantages and disadvantages that must be carefully weighed according to fracture type, patient profile and surgical goals.

In conclusion, dual plating demonstrated significantly higher initial construct rigidity and reduced displacement compared to TEN fixation, indicating superior early stability in midshaft clavicle fractures. These findings suggest that low-profile dual plating can enhance initial stability, making it a promising option for unstable midshaft clavicle fractures.

## Data Availability

No datasets were generated or analysed during the current study.
